# Successful Weaning From Veno-Venous Extracorporeal Membrane Oxygenation (VV-ECMO) After Initiation of Inhaled Epoprostenol in a Neonate With Refractory Persistent Pulmonary Hypertension of the Newborn (PPHN)

**DOI:** 10.7759/cureus.45595

**Published:** 2023-09-20

**Authors:** Irfan Shehzad, Ashish Banker, Bibhuti Das, Adil Humayun, Hale Wills, Muppala Raju, Niraj Vora

**Affiliations:** 1 Neonatology, Baylor Scott & White Health, Austin, USA; 2 Pediatric Cardiology, Baylor Scott & White Health, Temple, USA; 3 Neonatology, Baylor Scott & White Health, Temple, USA; 4 Pediatric Surgery, Baylor Scott & White Health, Temple, USA

**Keywords:** inhaled epoprostenol, extracorporeal membrane oxygenation, refractory pphn, meconium aspiration syndrome, neonate

## Abstract

Despite improvements in the medical management of persistent pulmonary hypertension of the newborn (PPHN), a significant number of patients persist with inadequate gas exchange and are treated with extracorporeal membrane oxygenation (ECMO). Prolonged time to weaning ECMO can increase mortality risk. Therefore, multiple therapies are utilized for pulmonary hypertension treatment, including pharmacotherapy with pulmonary vasodilators, to improve the prognosis of these critical patients. We report a case of a 37 2/7-week neonate with severe PPHN refractory to triple pulmonary vasodilator therapy (inhaled nitric oxide (iNO), sildenafil, and milrinone) and required veno-venous (VV)-ECMO support to improve oxygenation. Our patient was successfully weaned from ECMO after the addition of inhaled epoprostenol (iEPO) therapy. This report indicates that inhaled prostacyclin therapy effectively helps refractory PPHN patients off extracorporeal life support (ECLS) and should be considered a valuable treatment.

## Introduction

Persistent pulmonary hypertension of the newborn (PPHN) is a severe condition unique to the immediate neonatal period that occurs in approximately 10% of infants [1,2]. PPHN is characterized by failure to decrease the usually high fetal pulmonary vascular resistance (PVR) after birth, resulting in a marked decline in pulmonary blood flow (PBF) and, thus, oxygenation capacity of the lungs, leading to catastrophic clinical consequences for the neonate [3]. PPHN occurs due to birth asphyxia, meconium aspiration syndrome, respiratory distress syndrome, infections, congenital diaphragmatic hernia, and underlying heart or lung conditions [4,5]. PPHN is diagnosed based on clinical symptoms, physical examination, and diagnostic tests. These tests may include chest X-rays, blood gas analysis to assess oxygen and carbon dioxide levels, and echocardiography to evaluate right ventricle (RV) pressure with shunt direction across patent foramen ovale (PFO). Immediate treatment is crucial for PPHN. The primary goal for managing PPHN is to selectively decrease PVR and thereby improve oxygenation [6]. Treatment options may include supplemental oxygen, mechanical ventilation to support breathing, medications like inhaled nitric oxide (iNO) and Sildenafil to dilate blood vessels in the lungs, and high-frequency oscillatory ventilation (HFOV). Only iNO has been approved by the Food and Drug Administration (FDA) for treating PPHN, which improves oxygenation and reduces the need for extracorporeal membrane oxygenation (ECMO) in 60-70% of patients [7]. However, refractory PPHN occurs in 30-40% of iNO-treated neonates [8], and ECMO may be considered. Refractoriness depends on many factors, such as genetic background, gestational age, underlying conditions, and co-interventions; depending on these factors, only some patients can be considered candidates for ECMO [9]. Neonates with refractory PPHN have a high survival rate with ECMO support, but prolonged ECMO (> 7 days) is associated with a higher risk of complications and mortality [1]. Therefore, additional drugs are typically used in combination with iNO to effectively treat pulmonary hypertension and reduce the need for ECMO. Here, we report the case of a neonate with severe PPHN refractory to triple pulmonary vasodilator therapy (iNO, Sildenafil, and milrinone) and required HFOV. Veno-venous (VV)-ECMO support was used as all conventional interventions became ineffective. Subsequently, weaning off ECMO was difficult due to persistent high pulmonary vascular resistance. In our case, treatment with iEPO, an inhaled prostacyclin, facilitated successful weaning and decannulation from ECMO within 24 hours.

## Case presentation

**Figure 1:**
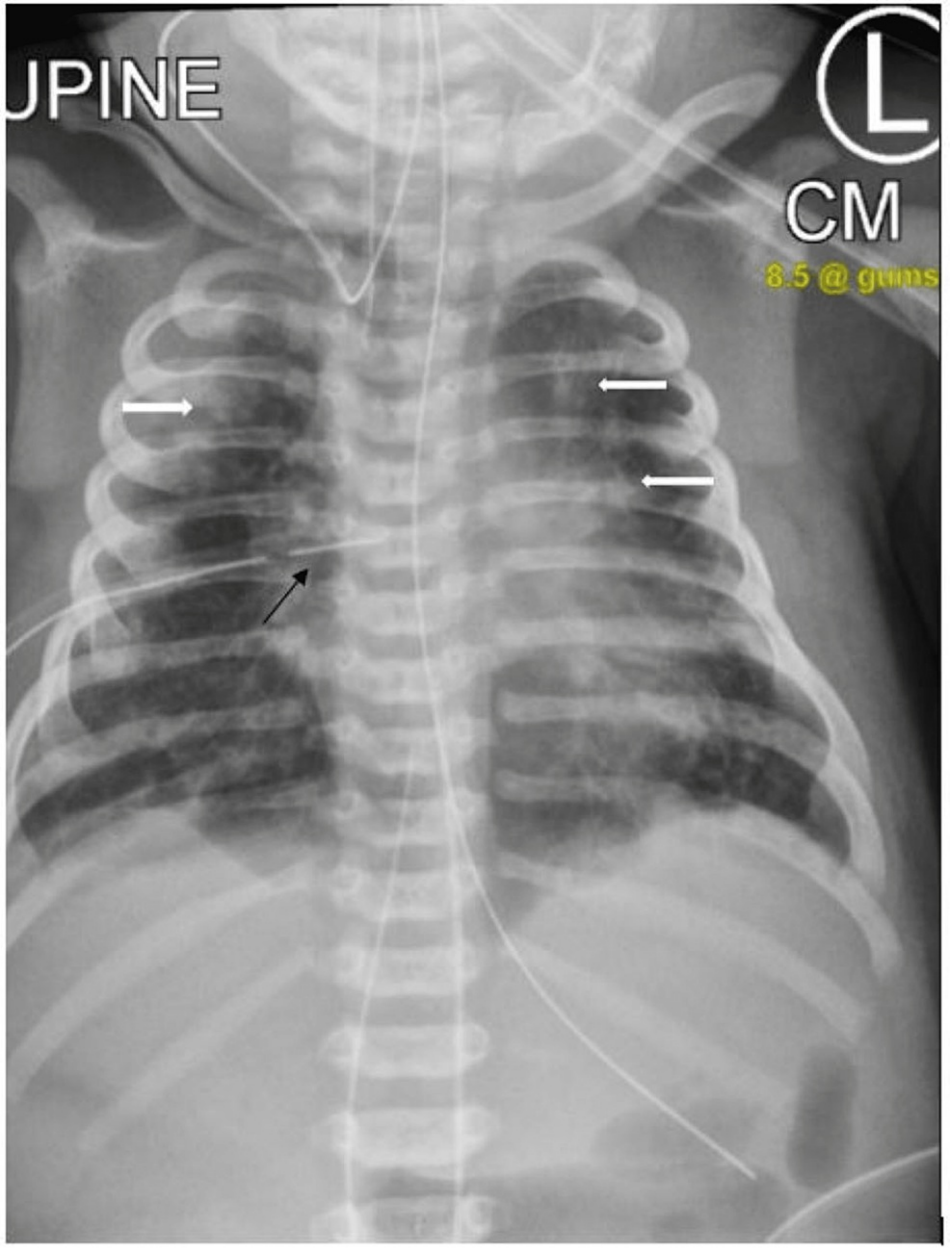
Chest X-ray, consistent with meconium aspiration syndrome (White arrows), shows resolved right tension pneumothorax, appropriate position of endotracheal tube, orogastric tube, umbilical lines, and right chest tube (Black arrow)

A female newborn, 37 2/7 weeks, 3080 grams, appropriate for gestational age, delivered vaginally at outside level 3 NICU to a 35-year-old G6P6A3L3 African American mother without prenatal care. There was a maternal history of opiates, cocaine, and tobacco use, with routine serology negative for infectious conditions except for unknown group B streptococcus (GBS) status without antibiotics prophylaxis treatment. The rupture of the membrane was 2 minutes before delivery, and APGAR scores at 1 minute and 5 minutes were 8 and 8, respectively. The patient had a positive urine drug screen with benzodiazepine and opiates and a positive meconium drug screen with cannabinoids. The patient aspirated thick meconium-stained amniotic fluid at delivery and immediately developed respiratory distress (SpO2 87% on 100% Fio2), requiring CPAP and then intubation at 10 minutes of life in the delivery room. The patient was transferred to NICU; surfactant was administered and started on continuous mandatory ventilation (CMV). The patient’s chest X-ray (Figure 1) showed bilateral pulmonary opacities consistent with meconium aspiration syndrome. The initial oxygenation index [OI = (FiO₂ × Mean Airway Pressure) / PaO₂] was 23 and PCO2 was 57 mmHg. A blood culture was drawn, and empiric IV ampicillin and gentamicin were started. The patient was transferred to our level 4 NICU after 4 hours of life (HOL). At 5 HOL, a right chest tube was inserted for moderate-size tension pneumothorax.

**Figure 2:**
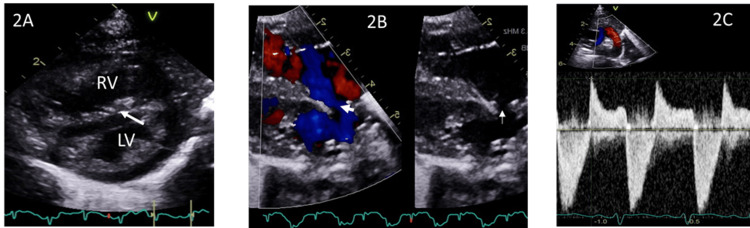
Pre-ECMO. (A) Ventricular septum is flat during diastole (arrow pointing at the septum), (B) Predominantly right-to-left shunt across patent foramen ovale (arrow), (C) Bidirectional shunt across the patent ductus arteriosus.

Echocardiogram showed RV is dilated due to severe PPHN (supra-systemic pulmonary artery pressure) evidenced by both systolic and diastolic flattening of the ventricular septum (Figure 2A) with mainly right to left shunt shunting across PFO (Figure 2B), a large patent ductus arteriosus (PDA) with bidirectional shunting (Figure 2C), but normal biventricular systolic function.

The ECMO team was alerted. The initial treatment for PPHN was high-frequency oscillatory ventilation (HFOV) for the optimization of oxygenation and ventilation, iNO (20 PPM), milrinone (0.5mcg/kg/min), enteral sildenafil (2mg/kg/q6h) to decrease PVR. Dexmedetomidine, morphine, and vecuronium drips were initiated for goals of sedation and analgesia to N-PASS-2 (Neonatal Pain, Agitation and Sedation Scale) and paralysis to ¼ twitches to prevent reactive pulmonary hypertension crises. In addition, the patient developed low cardiac output syndrome requiring additional inotropes (dopamine and norepinephrine) and was titrated to age-appropriate mean arterial pressure (MAP). Hydrocortisone was initiated as per protocol. Despite these measures, by 15 hours of life (HOL), the oxygenation index (OI) had increased to 88. The patient was cannulated for VV ECMO with a 13 Fr bi-caval Avalon cannula (Getinge AB, Göteborg, Sweden) in the right jugular vein with echocardiogram confirmation of the correct position of the return jet. Initial ECMO settings were pump flow 120 ml/kg/min (380 ml/min), sweep gas flow 0.25 L/min, 2000 RPM, and heparin infusion rate 30 units/hour [goal activated partial thrombosis time (APTT) of 2-3 x normal]. Since the patient had excellent lung recruitment on HFOV, it was decided to continue with HFOV with Hertz 12, amplitude 30, and mean airway pressure 15 to maintain while resting the lungs. Per institutional protocol, only colloids were used during the initial adjustment to ECMO, and crystalloid boluses were avoided. Coagulation functions were monitored with aPTT, ACT (Activated Clotting Time), and TEG 6s (Haemonetics, Boston, MA, USA) every 4 hours until heparin infusion resulted in goal aPTT. Day of life (DOL) 3/ECMO 2, inotropes were weaned and stopped, and 20ml/kg/day trophic feed was started. DOL 4/ECMO 3, left pleural effusion was noted, which resolved with intermittent furosemide and low renal dose (3 mcg/kg/min) dopamine. DOL 5/ECMO 4, a second dose of surfactant, was administered to alleviate the persistent surfactant deficiency caused by surfactant inactivation by meconium and improve oxygenation; MAP was also increased on HFOV to facilitate better oxygenation and ventilation. Sepsis workup, including blood culture, urine culture, respiratory viral panel, and limited TORCH (Toxoplasma, CMV, HSV PCR) screening, were all returned negative. DOL 7/ECMO 6, FiO2 weaned to 60% was tolerated. From DOL 7-10/ECMO 6-9, iNO was weaned slowly and then stopped. Daily serial echocardiogram from DOL 3-11/ECMO 2-10 showed closed PDA, but there was persistent flattening of the interventricular septum and dilated right atrium and right ventricle. Overall, ventricular function remained normal while our patient was on continuous milrinone infusion and ECMO support. The tricuspid jet could not be used for the right ventricular systolic pressure (RVSP) estimate due to the mode of ECMO cannulation.

**Figure 3:**
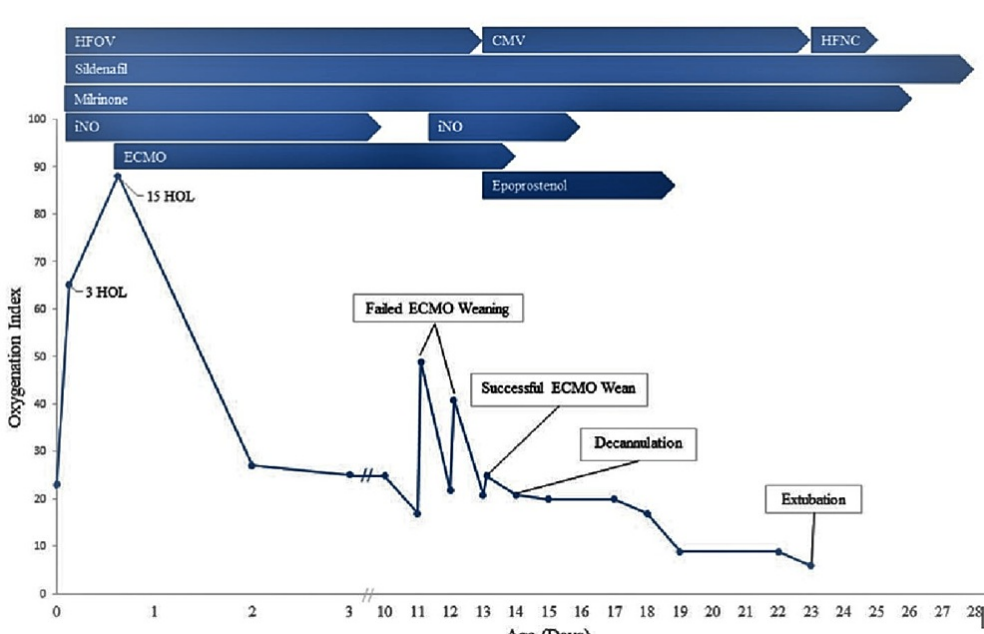
Oxygenation index over time. Blue arrows represent initiation to the cessation of respiratory support, pulmonary vasodilators, and ECMO. HOL: Hours of life; HFOV: High-frequency oscillatory ventilation; CMV: Continuous mandatory ventilation; HFNC: High-flow nasal cannula; iNO: Inhaled nitric oxide; ECMO: Extra-corporeal membrane oxygenation.

Trial off ECMO sweep was attempted on DOL 11-12/ECMO 10-11. Every time the patient started to desaturate to mid-70s% within 30 minutes of turning off the sweep gas, OI increased from < 20 to > 40 (Figure 3). An echocardiogram performed before and during trialing off ECMO also showed a worsening of PPHN. The patient recovered quickly and started to saturate > 95% after the sweep gas was turned on. The patient was switched to continuous mandatory ventilation, iNO was restarted at 20 PPM, dexamethasone was given per DART protocol [10], and a low dose of norepinephrine (0.1mcg/kg/min) was added to decrease pulmonary/systemic artery pressure ratio and improve cardiac performance [11]. Still, the patient continued to fail weaning trials from ECMO. On DOL 13/ECMO 12, iEPO (Veletri) was started at 20 ng/kg/min and stepwise increased by 10 ng/kg/min every 30 minutes to a maximum of 50 ng/kg/min dose as per protocol [12]. There was a significant clinical and echocardiographic improvement in PPHN within a few hours of starting iEPO. The patient tolerated the trial off sweep gas and ECMO wean, continued to maintain SpO2 > 95%, and OI remained stable. The patient was successfully decannulated 22 hours after initiating iEPO on DOL 14/ECMO 13. After decannulation from ECMO, pulmonary vasodilators (iNO, iEPO, milrinone, and sildenafil) were weaned and stopped in descending order (Figure 3).

**Figure 4:**
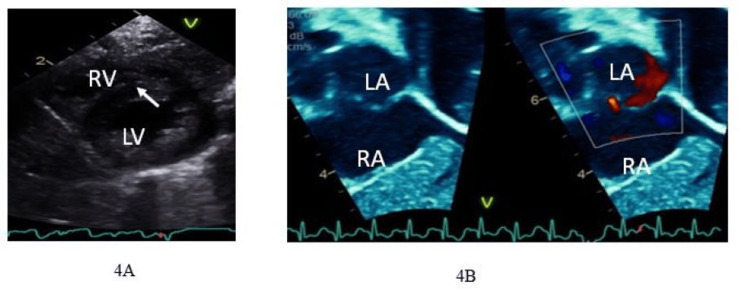
After decannulated from ECMO. (A) Ventricular septal position is normal. (B) Left-to-right shunting across the PFO.

The echocardiogram after ECMO was weaned off showed indirect measures of normal right ventricular systolic pressure, as seen from ventricular septal position (Figure 4A) and left-to-right shunt across the PFO (Figure 4B).

Antibiotics were stopped, and the chest tube was removed on DOL 19. Dexmedetomidine, morphine, and vecuronium were weaned slowly. The patient was extubated on DOL 23 to HFNC 4 L/min and weaned to room air on DOL 25. The patient developed neonatal abstinence syndrome, managed with oral methadone and clonidine. Brain MRI before discharge was also normal. The patient was able to tolerate full oral feeds and gained weight during hospitalization. The patient was discharged home on DOL 44 with foster parents due to the patient’s positive urine drug screen. The patient did well at the last general pediatric, pediatric cardiology, and NICU development clinic follow-up and was not on any pulmonary vasodilator therapy.

## Discussion

The current management of PPHN [13] includes mechanical ventilation, supplementary oxygen, blood pressure optimization, appropriate sedation, and pulmonary vasodilators, mainly iNO. Extracorporeal membrane oxygenation (ECMO) has been proposed for critical patients with PPHN refractory to conventional therapies [14]. The morbidity associated with ECMO in the neonatal population, particularly neurologic complications [14], and increased risk of morbidity and mortality with potential prolonged ECMO support makes it reasonable to consider the addition of advanced pulmonary vasodilator drugs as an option to limit ECMO exposure, especially in patients who are not candidates for ECMO (such as preterm neonates < 34 weeks, weighting < 2000 grams, neonates with evidence of major intracranial hemorrhage or lethal congenital anomalies) or to complement the pulmonary vasodilator effects of iNO to improve the outcome of infants with difficulty weaning from ECMO support. Lazar et al. reviewed the outcomes and pre-ECMO variables of increased mortality in neonates with PPHN receiving ECMO. In this cohort, neonates with PPHN receiving ECMO support for 7, 10, 14, and 21 days survived at rates of 88%, 78%, 55%, and 25%, respectively [1].

Four main categories of pulmonary vasodilators are available, such as prostacyclin analogs (iloprost, epoprostenol), phosphodiesterase type 5 inhibitors (sildenafil), phosphodiesterase type 3 inhibitors (milrinone), and endothelin receptor blockers (bosentan). These drugs are commonly used in adolescents and adults to treat pulmonary hypertension, but only a few reports have addressed their safety and efficacy in neonates [15]. These drugs act on four different pathways from iNO, and combination therapy can have synergistic effects [15]. They should be used in a case-by-case scenario in neonates with PPHN, considering their unique pharmacological features, safety, side effects, and PPHN pathobiology.

In an extremely acute setting like refractory PPHN, orally administered drugs such as bosentan and sildenafil are less suitable since they may need a longer time to reach an effective systemic distribution, and little to nothing is known about their intestinal absorption in neonates [16,17]. Intravenous formulation of Sildenafil is not always available and is frequently associated with clinically significant systemic hypotension [18]. Conversely, oral drugs can be helpful in the weaning phase and for maintenance therapy. The vasodilatory potency on the pulmonary vascular bed for sildenafil is significantly lower than that of the prostacyclin analog [19]. Milrinone, which can increase myocardial contractility and reduce afterload, is considered in case of impaired RV contractility and helps decrease pulmonary vascular impedance [15].

Prostacyclin (PGI2) is a naturally occurring prostanoid produced via arachidonic acid metabolism by vascular endothelial cells. PGI2 is an important mediator of pulmonary vasodilation and is known to play a crucial role during adaptation to extrauterine life [20]. Epoprostenol, the sodium salt of prostacyclin, is the first exogenous prostanoid used to treat pulmonary hypertension [21] and is available in IV and inhaled formulations. It stimulates the production of cyclic adenosine monophosphate (cAMP)-mediated vasodilation, resulting in pulmonary and systemic vasodilation. Epoprostenol is FDA-approved for treating pulmonary hypertension in adults [22]; however, it is important to note that the use of iEPO in neonates and children is considered off-label and should be carefully monitored for side effects such as hypotension, flushing, cyanosis, feeding intolerance, and pain.

In neonates, limited data exist regarding the efficacy of inhaled epoprostenol (iEPO) for treating PPHN. iEPO was evaluated by Brown et al. [23]; the authors reported a significant improvement in OI from 25.6 to 14.5 in 13 neonates with PPHN. A study by Ahmad et al. [5] demonstrated an apparent efficacy of IV epoprostenol in neonates with iNO-refractory PPHN, with improved OI, most notable in patients with meconium aspiration syndrome and infectious etiologies. This clinically significant response was seen within 4 hours of initiating IV epoprostenol, and the effect was sustained through 24 hours. For refractory PPHN, iEPO is favorable over the IV formulation due to fewer systemic side effects of hypotension and appears to be an alternative with similar efficacy and safety as iNO but at a reduced cost [24]. Moreover, when PPHN is secondary to a lung parenchymal disease such as respiratory distress syndrome or meconium aspiration syndrome, etc., iEPO will preferentially reach lung areas that have been recruited and therefore are well-ventilated. Thus, the risk of increasing intrapulmonary shunt and worsening the ventilation/perfusion mismatch is lower with iEPO [15]. This risk is less compelling in lung development disorders or pulmonary hypoplasia due to genetic anomalies [25]. Modern vibrating mesh nebulizers provide satisfactory drug delivery in continuous mandatory and high-frequency oscillatory ventilation. The same setting can also be efficaciously used during noninvasive ventilation [15]. Epoprostenol has a short half-life of 6.5-10 minutes, necessitating its continuous intravenously or inhaled delivery [26, 27]. Due to the short half-life of epoprostenol, response (if any) can be quickly detected without delaying other treatments and ECMO. Dosing of iEPO ranges from 10 to 50 ng/kg/min and may be titrated to clinical effect with reported significant improvement in PPHN at 30 ng/kg/min [26, 28].

Our reported patient showed unresponsiveness to triple pulmonary vasodilators (iNO, sildenafil and milrinone) and was placed on VV-ECMO support due to a continued increase in OI and worsening of end-organ function despite multiple inotropes and HFOV. This vasodilator refractory PPNH was also noted during the trial of ECMO, prompting a search for additional therapeutic options. Initiation of iEPO resulted in rapid resolution of PPHN, improved ventilation-perfusion matching, improved OI, and weaning from ECMO. Thus, the patient was successfully taken off ECMO within 24 hours of initiating iEPO without complications and had an excellent prognosis.

## Conclusions

A patient with refractory PPHN successfully came off ECMO within 24 hours of starting iEPO, leading to a favorable prognosis. This promising outcome suggests that administering inhaled prostacyclin early on can be an effective therapy in aiding the weaning-off process and reducing the time spent on ECLS for patients who have refractory PPHN. However, a more comprehensive study with a larger group of patients is necessary to investigate these findings further.
